# Assortative Mating by Ethnicity in Longevous Families

**DOI:** 10.3389/fgene.2017.00186

**Published:** 2017-11-21

**Authors:** Paola Sebastiani, Anastasia Gurinovich, Harold Bae, Stacy L. Andersen, Thomas T. Perls

**Affiliations:** ^1^Department of Biostatistics, Boston University School of Public Health, Boston, MA, United States; ^2^Bioinformatics Program, Boston University, Boston, MA, United States; ^3^College of Public Health and Human Sciences, Oregon State University, Corvallis, OR, United States; ^4^Geriatrics Section, Department of Medicine, Boston University School of Medicine and Boston Medical Center, Boston, MA, United States

**Keywords:** extreme longevity, genome-wide principal component analysis, endogamy, exogamy, ethnicity

## Abstract

Recent work shows strong evidence of ancestry-based assortative mating in spouse pairs of the older generation of the Framingham Heart Study. Here, we extend this analysis to two studies of human longevity: the Long Life Family Study (LLFS), and the New England Centenarian Study (NECS). In the LLFS, we identified 890 spouse pairs spanning two generations, while in the NECS we used data from 102 spouse pairs including offspring of centenarians. We used principal components of genome-wide genotype data to demonstrate strong evidence of ancestry-based assortative mating in spouse pairs of the older generation and also confirm the decreasing trend of endogamy in more recent generations. These findings in studies of human longevity suggest that spouses marrying into longevous families may not be powerful controls for genetic association studies, and that there may be important ethnicity-specific, genetic influences and/or gene–environment interactions that influence extreme survival in old generations. In addition, the decreasing trend of genetic similarity of more recent generations might have ramifications for the incidence of homozygous rare variants necessary for survival to the most extreme ages.

## Introduction

One of the surprising findings that has emerged from the Long Life Family Study (LLFS) – a longitudinal family-based study of longevity and healthy aging – is that spouses of members of longevous families tend to be healthier than average. In Sebastiani et al. (2013), we showed that spouses of LLFS probands and their siblings have delayed onset of morbidity compared to controls enrolled in the New England Centenarian Study (NECS). A recent article by Pedersen et al. (2017) showed that the mortality of spouses marrying into the longevity-enriched families of LLFS was substantially lower than the mortality in a background population matched by birth year and sex. One obvious explanation for the spouses being so healthy is that they share the same environment as the participants from longevous families. Another contributing explanation, we hypothesize, is assortative mating – a pattern of sexual selection in which individuals with similar phenotype(s) and hence similar genotypes mate more frequently than randomly. It has been also proposed that assortative mating of members from longevous families could maintain longevity across generations by increasing the likelihood of transmission of rare variants with a recessive effect (Govindaraju, 2015). However, we are not aware of genetic studies supporting this latter hypothesis.

Genetic evidence of assortative mating in many human traits and disease has been demonstrated using a variety of analyses. For example the recent work by Robinson et al. (2017) used sophisticate regression analyses of genome-wide single nucleotide polymorphisms (SNPs) to show evidence of assortative mating in several traits including heights, BMI, and other metabolic traits using the UK Biobank study. The recent publication by Sebro et al. (2017) describes a simple method to measure the degree of ancestry-based genetic similarity of spouse-pairs using a summary of genome-wide genotype data. The method’s assumption is that the genetic background of an individual can be well-characterized by few principal components computed from the principal component analysis (PCA) of genome-wide genotype data (Patterson et al., 2006), and therefore ancestry-related genetic similarity of a set of spouse pairs can be described by the correlation between their genome-wide principal components. This method was applied to spouse pairs in the two oldest generations of the Framingham Heart Study (Tsao and Vasan, 2015), and provided strong evidence of assortative mating that declines with generation. The authors noted the lack of replication of their finding in independent studies and we propose here to replicate these analyses and test for evidence of assortative mating among members of longevous families and their spouses using genome-wide genotype data from 890 spouse pairs enrolled in LLFS and 105 spouse pairs enrolled in the NECS (Sebastiani and Perls, 2012). We also examine whether the degree of genetic similarity between spouse pairs decreases with successive generations.

## Materials and Methods

### Study Populations

The LLFS is a family-based study of healthy aging and longevity that enrolled approximately 5,000 individuals belonging to 550 families selected for familial longevity. The LLFS recruited participants from the United States and Denmark. Potential probands were recruited based in part on their Family Longevity Selection Score (FLoSS) that quantifies the degree of familial longevity using sex and birth-year cohort survival probabilities of the proband and their siblings (Sebastiani et al., 2009). Eligibility of sibships for the study was based on a FLoSS score > 7 and having at least one living sibling and one offspring willing to be enrolled in the study. Data collected on study participants are described in Newman et al. (2011) and Sebastiani et al. (2017b). Genome-wide genotype data were generated using the Illumina 2.0 million SNP array as previously described (Bae et al., 2013). Data are available via dbGaP (Study Accession: phs000397.v1.p1). The NECS is a study of centenarians which in about a third of the cases includes long-lived siblings, their offspring, offspring spouses and additional unrelated controls, all primarily from North America (Sebastiani and Perls, 2012). Data collected at enrollment and through annual follow-ups are described in Andersen et al. (2012) and Sebastiani and Perls (2012). The NECS has very few spouse pairs in the proband generation by virtue of the extreme age of the probands at the time of enrollment and so we could only study spousal pairs in the offspring generation.

Genome-wide genotype data were generated using Illumina SNP arrays (Sebastiani et al., 2012). All subjects provided informed consent approved by the Boston University Medical Campus IRB. Additional study details are available from http://www.bumc.bu.edu/centenarian/.

### Statistical Analysis

**FIGURE 1:**
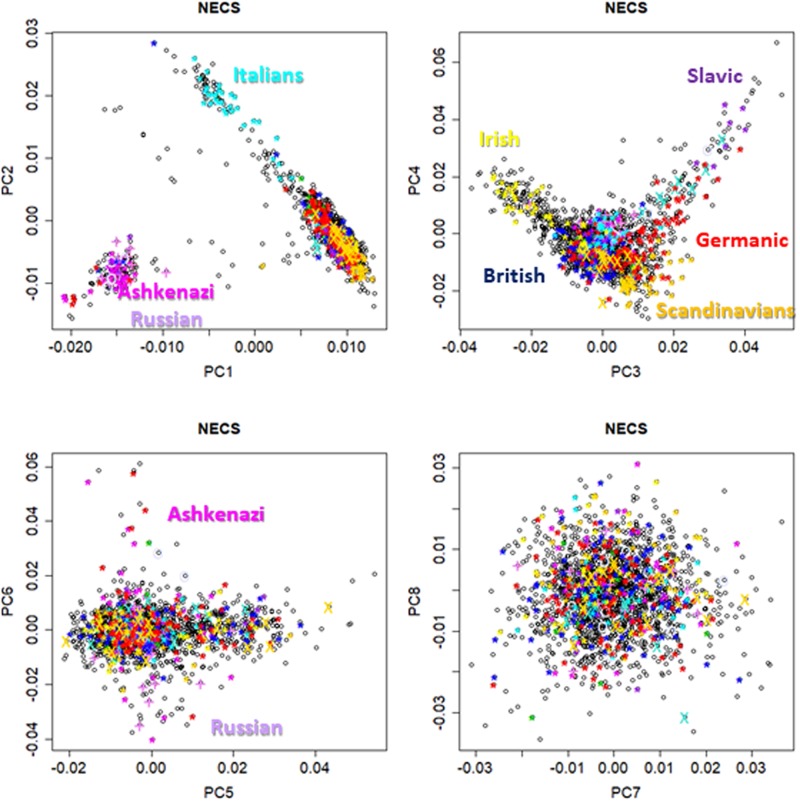
Pairwise scatter plots of the first eight principal components from genome-wide principal component analysis (PCA) in participants from the New England Centenarian Study (NECS). European ethnicities were defined based on parents and grand-parents mother tongue, and birth place.

**FIGURE 2:**
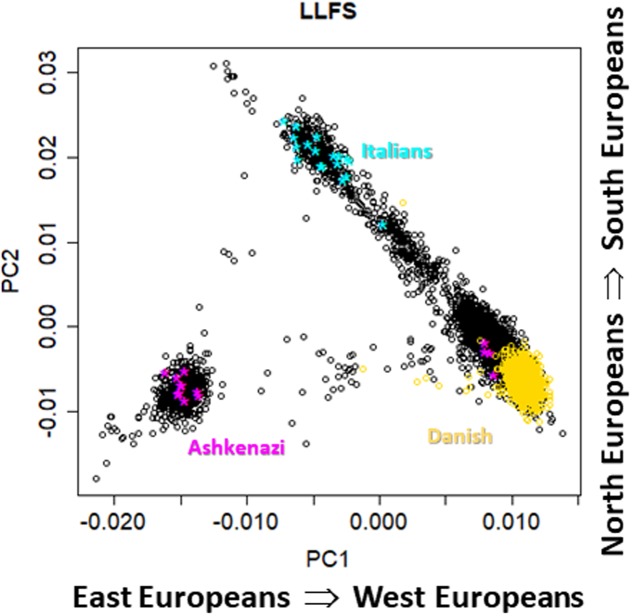
Scatter plot of the first two principal components from genome-wide principal components analysis in 4,630 LLFS participants. Gold: participants enrolled at the Danish field center; cyan: participants born in Italy; magenta: participants born in Poland or Russia.

We conducted a genome-wide PCA with the program EIGENSOFT (Price et al., 2006, 2008) using a data set of multiple European ethnicities including all of the LLFS and NECS participants of white race. The data included approximately 70,000 independent and common SNPs that were selected to be common to the two studies, with a call rate > 0.95 and minor allele frequency > 0.05. SNPs in strong LD were removed using the program PLINK with a SNP window of 50 and sliding window of five SNPs and we removed one SNP from each pair of SNPs with *r*^2^ > 0.30 (see additional details in Solovieff et al., 2010; Sebastiani et al., 2012, 2017a). We selected for analysis the first eight principal components after visual inspection of the scree plot (see Supplementary Figure S1) and these principal components explained 68% of the variance captured by the first 30 principal components. We characterized European ethnicities by using ethno-linguistic groups that were labeled using known birth-places and mother tongue of study participants or their parents when available (**Figures 1**, **2** and Supplementary Figure S2). This approach is consistent with other investigations of European ethnicities (Nelis et al., 2009). Spouse pairs in NECS and LLFS were identified by in-common children in linkage files, and the genetic similarities of spouse pairs was measured via Person correlation of the first six principal components (**Table 1**). For comparison, we selected LLFS offspring of East European ethnicity (PC1 < 0, PC2 < 0) or North European ethnicity (PC1 > 0, PC2 < 0) and generated heterosexual pairs by randomly selecting spouses with the same ethnicity. This random assortment was repeated 1000 times, and Pearson correlations of PC1 and PC2 in the random pairs were summarized with mean and 95% quantile intervals. Analyses were conducted using R v3.3.

**Table 1:** Summary statistics of spouse pairs.

	LLFS		LLFS (no DK)		NECS
	Proband	Offspring	Proband	Offspring	Offspring
N spouse pairs	155	753	130	373	102
Birth year	1920 (1909–1944)	1945 (1919–1970)	1920 (1909–1944)	1945 (1919–1970)	1932 (1919–1949)
Age	93 (72–103)	71 (45–97)	93 (72–103)	71 (45–97)	84 (67–97)
Cor PC1	0.79 (0.71; 0.84)	0.71 (0.68; 0.75)	0.76 (0.67; 0.82)	0.63 (0.57; 0.69)	0.67 (0.55;0.77)
Cor PC2	0.70 (0.59; 0.76)	0.40 (0.33; 0.45)	0.66 (0.55; 0.75)	0.26 (0.16; 0.35)	0.20 (0.01; 0.38)
Cor PC3	0.57 (0.46; 0.67)	0.44 (0.38; 0.50)	0.54 (0.40; 0.65)	0.24 (0.14; 0.34)	0.11 (-0.09; 0.30)
Cor PC4	0.68 (0.59; 0.76)	0.65 (0.61; 0.69)	0.56 (0.43; 0.67)	0.32 (0.22; 0.41)	0.02 (-0.18; 0.21)
Cor PC5	0.16 (0.01; 0.32)	0.02 (-0.06; 0.09)	0.19 (0.02; 0.35)	-0.01 (-0.12; 0.09)	0.11 (-0.09; 0.30)
Cor PC6	0.08 (-0.08; 0.23)	0.02 (-0.04; 0.10)	0.06 (-0.11; 0.24)	0.01 (-0.09; 0.11)	0.05 (-0.15; 0.24)

*LLFS, Long Life Family Study; LLFS (no DK), Long Life Family Study participants excluding participants enrolled at the Denmark field center. NECS, New England Centenarian Study. Birth year summaries are median and range of all individuals in each set of pairs*.

## Results

**Figure 1** shows pairwise scatter plots of the first eight principal components in NECS participants for whom we had information about European ancestry based on birth place and parent’s and grand-parent’s mother tongue (*n* = 1,979) (Sebastiani and Perls, 2012). The plots are consistent with previous published analyses which showed that the first four genome-wide principal components can capture major axes of variation of European ethnicities (Reich et al., 2008; Tian et al., 2009; Solovieff et al., 2010; Sebastiani et al., 2012), when the PCA analysis is limited to Whites. The first two principal components (PC1 and PC2) capture ethnic variations from East to West and North to South Europe. The next two principal components (PC3 and PC4) differentiate between ethnic groups from North Europe, with Irish, Scandinavian and Slavic ethnicities that are represented at the three corners of the plot. The next two principal components (PC5 and PC6) distinguish East Europeans of Ashkenazi Jewish descent from Russians. Principal components PC7 and PC8 do not seem to inform further about ethnicity. **Figure 2** shows the scatter plot of PC1 and PC2 in 4,630 LLFS participants and highlights LLFS participants of known European ethnicity based on birth place or enrollment center. The represented ethnicities are consistent with the data in **Figure 1**. Plots of higher order PCs also show patterns similar to those in **Figure 1**.

**FIGURE 3:**
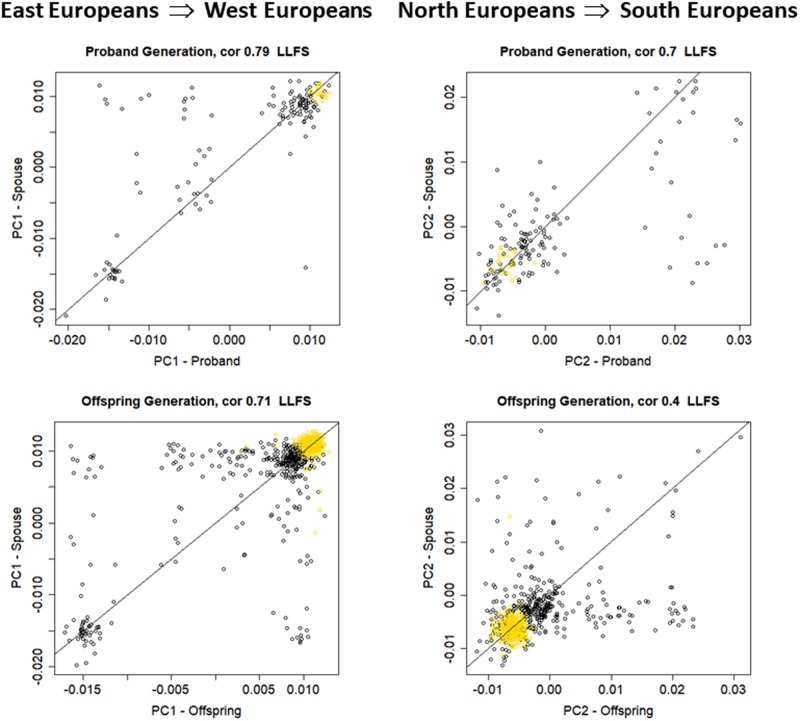
Scatter plot of PC1 and PC2 in the LLFS spouse pairs. Top: proband generation (*N* = 155). Bottom: offspring generation (*N* = 735). Gold: participants enrolled at the Danish field center.

Among the 4,630 LLFS participants with genetic data, we identified 155 spouse pairs in the proband generation and 735 spouse pairs in the offspring generation (**Table 1**). The top panel of **Figure 3** shows the scatter plots between PC1 (*r* = 0.79, 95% CI = 0.72; 0.84, *p*-value < 2.2E-16) and between PC2 (*r* = 0.70, 95% CI = 0.61; 0.77, *p*-value < 2.2E-16) in the 155 spouse pairs of the proband generation. The analysis shows strong within-ethnicity marriage with only 12 of the 155 pairs representing marriages between East and North West European ethnicities (*n* = 5), and North and South Europeans (*n* = 7). The bottom panel of **Figure 3** shows the same scatter plots in the 735 spouse pairs of the offspring generation. While the correlation of PC1 in spouse pairs of the offspring generation (*r* = 0.71, 95% CI = 0.68; 0.75, *p*-value < 2.2 E-16) is only slightly lower than in the proband generation, the correlation of PC2 is substantially lower (*r* = 0.40, 95% CI = 0.33; 0.45, *p*-value < 2.2E-16) and suggests increased number of marriages between East and North–West Europeans, and North–West and South–West Europeans. We also conducted a subset analysis excluding all participants enrolled at the Danish field center of LLFS, to examine whether the enrollment in a specific European region could increase the ancestry-based genetic similarity. This analysis shows minor changes in the correlations of PC1—PC6 in the proband generation, and a more substantial decrease of correlation in the offspring generation. However, the correlation of PC1 through PC4 in the 373 spouse pairs enrolled in the United States remains statistically significant.

**FIGURE 4:**
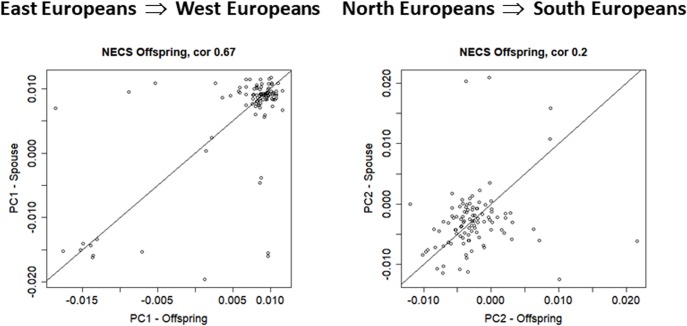
Scatter plot of PC1 and PC2 in the NECS spouse pairs (*N* = 102).

In NECS, we identified 102 pairs in the offspring of centenarians and their spouses. **Figure 4** displays scatter plots of PC1 and PC2 of these pairs and the large correlation of PC1 (*r* = 0.67, 95% CI = 0.55; 0.77, *p*-value = 8.572e-15) is consistent with a large proportion of marriages within the main gradient of European ethnicities. The within-spouse correlation of PC2 (*r* = 0.20, 95%CI = 0.01; 0.38, *p*-value = 0.04142) is lower compared to LLFS offspring but remains significantly different from 0 and is consistent with the observation in the LLFS offspring generation, again suggesting decreased ethnicity homogeneity among married couples among offspring.

We randomly selected individuals from the pool of spouses in LLFS, and generated random heterosexual pairs, with each pair including one of the 635 offspring of East or North European ethnicity, and a randomly chosen spouse from the same ethnic group. In 1000 replications, the mean correlation of PC1 in the 635 pairs was 0.30, while the mean correlation of PC2 was 0.07.

## Discussion

We estimated the degree of ancestry-related genetic similarity in spouse pairs from two studies of healthy aging and longevity, the LLFS and the NECS. Our analysis showed significant positive correlation between PC1 of spouse pairs that ranged from 0.79 for LLFS proband generation (median birth year: 1920), to 0.67 for NECS offspring generation (median birth year: 1932), and to 0.63 for LLFS offspring generation (median birth year: 1945). The analysis also showed significant positive correlation of PC2 that ranged from 0.70 for spouses of LLFS proband generation to 0.20 for spouses of NECS offspring generation. Since PC1 represents the gradient from Eastern to Western European ethnicities, and PC2 provides a finer separation of Northern European ethnicities, the large correlations of PC1 and PC2 in spouse pairs of the oldest generation of LLFS are consistent with strong endogamy of the pre-World War II generations. The decreasing correlation of PC1 and PC2 in offspring spouses of both LLFS and NECS is consistent with decreased endogamy (increased exogamy) of later generations.

These results are consistent with the data in Sebro et al. (2017) that showed a 0.73 correlation of PC1 (95% CI 0.63; 0.80) and 0.80 correlation of PC2 (95% CI 0.72; 0.85) in spouse pairs of the original generation enrolled in the Framingham Heart Study, and smaller correlations of PC1 and PC2 in spouse pairs of the offspring generation [PC1: *r* = 0.38; 95% CI: (0.32, 0.44); PC2: *r* = 0.45; 95% CI (0.39, 0.51)]. We observed a larger correlation of PC1 and a smaller correlation of PC2 in both LLFS generations and the NECS offspring generations compared to spouse pairs of the Framingham Heart Study. This result could be attributable to a greater ethnic heterogeneity of LLFS and NECS participants. Despite the name, the NECS enrolls participants from throughout North America, and other English speaking countries and for example 8% of participants are of Ashkenazi Jewish descent or Russian ancestry compared to the 5% prevalence in the town of Framingham reported in Sebro’s work. Similarly, approximately 12% of all LLFS participants are of Ashkenazi Jewish descent. Our analysis showed that a substantial proportion of marriages between individuals from different ethnic groups involves study participants of Ashkenazi Jewish descent and could be the reason for the weaker correlation of PC2 that we observed in our data.

The high degree of genetic similarities of spouse pairs, particularly for older generations, has several implications to genetic studies that are comprehensively described in Sebro et al. (2017). In the context of longevity, our analysis provides evidence for ancestry-related assortative mating also in families selected for longevity. A link between assortative mating and human longevity was conjectured in early 1900 studies of eugenics (Lund and Anderson, 1914), based on the fact that longevity was a trait that families could be proud of and families with this trait could form alliances to produce strong progeny. Studies of ethnically isolated groups characterized by high rates of endogamy in Sardinia, Southern Italy, Okinawa, and Ashkenazi Jewish ancestry further provide the possibility that assortative mating is one of the possible mechanisms of human longevity (Caselli et al., 2006; Govindaraju, 2015). More recently, analyses of couples from the UK Biobank has shown that there is assortment for longevity and disease (Rawlik et al., unpublished) in addition to a variety of traits (Robinson et al., 2017). Noticeably, the average correlations of PC1 and PC2 in artificially created spouse pairs including LLFS offspring and randomly selected spouses with matching ethnicity were substantially lower than the correlation of the real spouse pairs (PC1: 0.30 versus 0.71; PC2: 0.07 versus 0.4). The higher degree of genetic correlation observed in this set of spouses compared to ethnically matched spouse pairs provides additional evidence of an association between longevity and assortative mating.

While our analysis does not show that assortative mating is one of the selection mechanisms for longevity, it provides support to this hypothesis and has implications that are specific to studies of longevity. It is possible that there are important ethnicity-specific genetic influences and/or gene–environment interactions that influence extreme survival. For example, alleles of *APOE* vary substantially by race and ethnicity and it has been conjectured that diet can modify the effect of the allele ε_4_ (Corbo and Scacchi, 1999). Evidence of assortative mating suggests that spouses of members of longevous families may not be powerful controls in genetic association studies because they may be too similar to their partner and inclusion of unrelated controls could increase statistical power (Sebastiani et al., 2012, 2017a). Past studies have shown that inclusion of related individuals has limited impact on the statistical power of genetic association studies (Visscher et al., 2008), and the extent of the impact of assortative mating in genetic association studies of human longevity needs to be investigated.

An additional observation that is relevant to extreme human longevity is the decreasing trend of the degree of genetic similarity with younger and younger generations in the United States. We and others have shown that rare, recessive genotypes are associated with extreme human longevity (Schachter et al., 1994; Barzilai et al., 2003; Tazearslan et al., 2012; Sebastiani et al., 2017a) and therefore the decrease of endogamy could lead to a decreased incidence of these longevity promoting variants in the population and to a future contraction in prevalence of extremely long-lived individuals where such variants play deterministic roles in extreme survival. Recently, Dong et al. (2016) used data showing a reduction in maximum lifespan of supercentenarians in the last decade to conclude that there is a biological limit to human life-span. While this analysis has limitations as noted in Brown et al. (2017); de Beer et al. (2017), Hughes and Hekimi (2017); Lenart and Vaupel (2017), and Rozing et al. (2017), our results suggest an alternative hypothesis that increased population mobility and reduction of endogamy may contribute to more limited opportunities for transmission of rare recessive variants in the population thus limiting the opportunity to observe someone extending human lifespan, which has plateaued since 1997.

## Author Contributions

PS and TP formulated the research question, planned the analysis and drafted the manuscript. HB and AG contributed to the data analysis. SA contributed to patients enrollment, collection of data, and drafting the manuscript.

## Conflict of Interest Statement

The authors declare that the research was conducted in the absence of any commercial or financial relationships that could be construed as a potential conflict of interest.
