# Supplementary material for: Assortative Mating by Ethnicity in Longevous Families
Source: Front Genet. 2017 Nov 21;8:186. doi: 10.3389/fgene.2017.00186 (PMC5702482; doi:10.3389/fgene.2017.00186)

Supplement Material to

Assortative Mating By Ethnicity In Longevous Families

Paola Sebastiani[1], Anastasia Gurinovich[2], Harold Bae[3], Stacy L Andersen[4], Thomas T Perls[4]

Figure S1. Scree plot of the principal components generated in the data aggregated from the New England Centenarian Study and the Long Life Family Study.

Figure S2. Map of European ethnicities and language groups.

Figure S1. Scree plot of the principal components generated in the data aggregated from the New England Centenarian Study and the Long Life Family Study. Y-axis=eigenvalues; x-axis=Principal components.

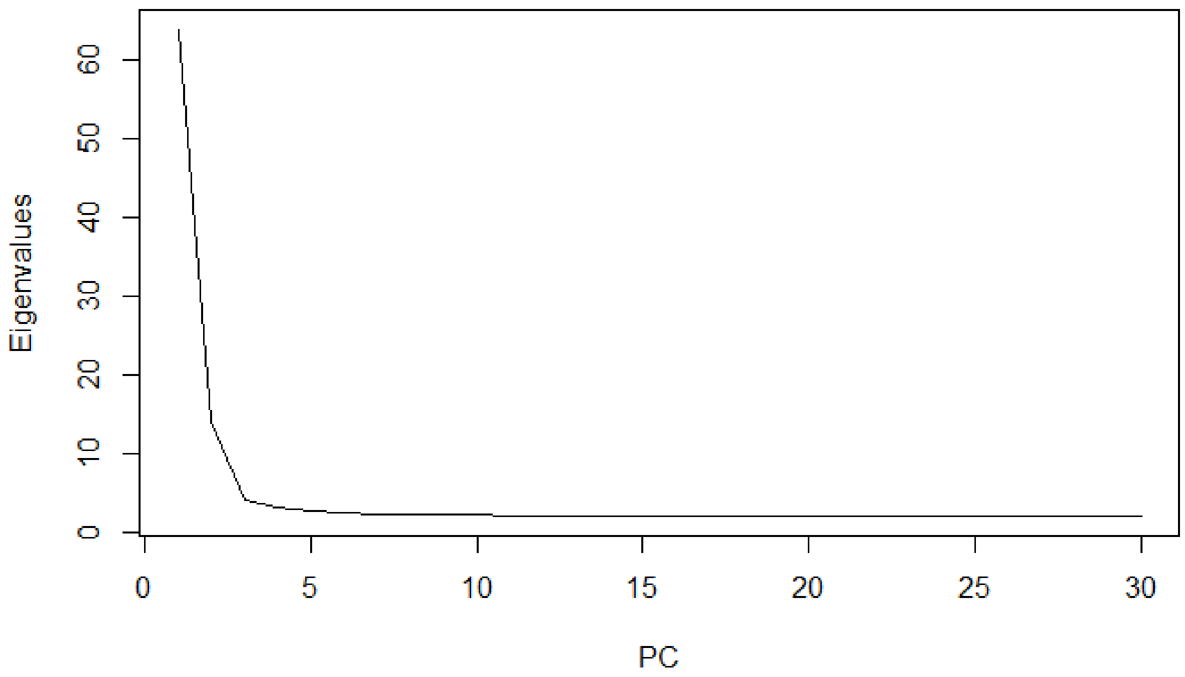

Figure S2. Map of European ethnicities and language groups.

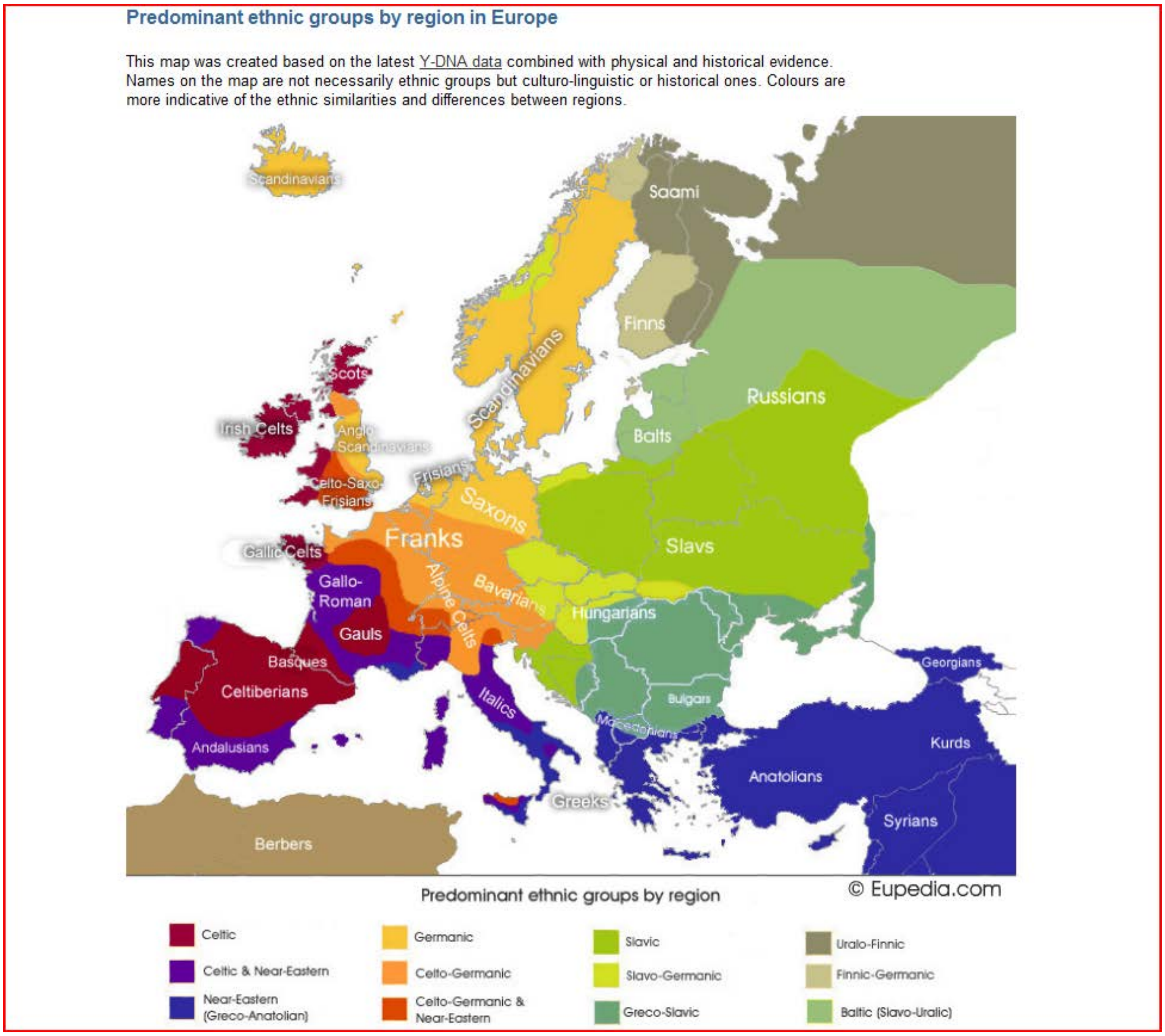

Supplement: Supplementary file 1 [file Image_1.PDF]
